# Molecular basis of an atypical dsDNA 5mC/6mA bifunctional dioxygenase CcTet from *Coprinopsis cinerea* in catalyzing dsDNA 5mC demethylation

**DOI:** 10.1093/nar/gkae066

**Published:** 2024-02-07

**Authors:** Lin Zhang, Yajuan Mu, Tingting Li, Jingyan Hu, Houwen Lin, Liang Zhang

**Affiliations:** Department of Pharmacology and Chemical Biology, State Key Laboratory of Systems Medicine for Cancer, Shanghai Jiao Tong University School of Medicine, Shanghai 200025, China; Department of Pharmacology and Chemical Biology, State Key Laboratory of Systems Medicine for Cancer, Shanghai Jiao Tong University School of Medicine, Shanghai 200025, China; Department of Pharmacology and Chemical Biology, State Key Laboratory of Systems Medicine for Cancer, Shanghai Jiao Tong University School of Medicine, Shanghai 200025, China; Department of Pharmacology and Chemical Biology, State Key Laboratory of Systems Medicine for Cancer, Shanghai Jiao Tong University School of Medicine, Shanghai 200025, China; Research Centre for Marine Drugs, State Key Laboratory of Oncogene and Related Genes, Department of Pharmacy, Ren Ji Hospital, School of Medicine, Shanghai Jiao Tong University, Shanghai 200127, China; Institute of Marine Biomedicine, Shenzhen Polytechnic, Shenzhen 518055, China; Department of Pharmacology and Chemical Biology, State Key Laboratory of Systems Medicine for Cancer, Shanghai Jiao Tong University School of Medicine, Shanghai 200025, China

## Abstract

The eukaryotic epigenetic modifications 5-methyldeoxycytosine (5mC) and *N*^6^-methyldeoxyadenine (6mA) have indispensable regulatory roles in gene expression and embryonic development. We recently identified an atypical bifunctional dioxygenase CcTet from *Coprinopsis cinerea* that works on both 5mC and 6mA demethylation. The nonconserved residues Gly331 and Asp337 of CcTet facilitate 6mA accommodation, while D337F unexpectedly abolishes 5mC oxidation activity without interfering 6mA demethylation, indicating a prominent distinct but unclear 5mC oxidation mechanism to the conventional Tet enzymes. Here, we assessed the molecular mechanism of CcTet in catalyzing 5mC oxidation by representing the crystal structure of CcTet–5mC-dsDNA complex. We identified the distinct mechanism by which CcTet recognizes 5mC-dsDNA compared to 6mA-dsDNA substrate. Moreover, Asp337 was found to have a central role in compensating for the loss of a critical 5mC-stablizing H-bond observed in conventional Tet enzymes, and stabilizes 5mC and subsequent intermediates through an H-bond with the *N*^4^ atom of the substrates. These findings improve our understanding of Tet enzyme functions in the dsDNA 5mC and 6mA demethylation pathways, and provide useful information for future discovery of small molecular probes targeting Tet enzymes in DNA active demethylation processes.

## Graphical Abstract

**Graphical Abstract:**
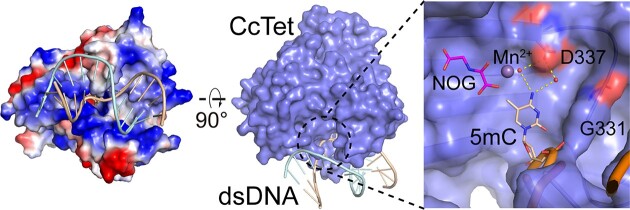

## Introduction

DNA methylation (5-methyldeoxycytosine, 5mC) is the most abundant epigenetic modification in eukaryotes (∼1–4% of the total cytosines) (1,2). The methyl group adduct on the 5-position of the pyrimidine ring interferes in the recognition by transcription factors, significantly altering subsequent gene expression and transcription, and thereby plays vital roles in genetic and cellular processes, including gene expression, genomic imprinting, X-chromosome inactivation, embryonic development and epigenetic inheritance (1,3). Besides 5mC, *N*^6^-methyldeoxyadenine (6mA) is a recently identified eukaryotic epigenetic modification on duplex DNA (dsDNA), which has been found in the genomes of numerous eukaryotic species, including green algae (4), ciliates (5,6), worms (7), fungi (8) and plants (9,10). Notably, 6mA was also identified in mammalian mitochondrial and nuclear genomes, and may play epigenetic roles in transposable element expression, cell development and mitochondrial transcription (11–17), although such findings were dismissed as technical artifacts in later studies (18–20).

**Figure 1.**
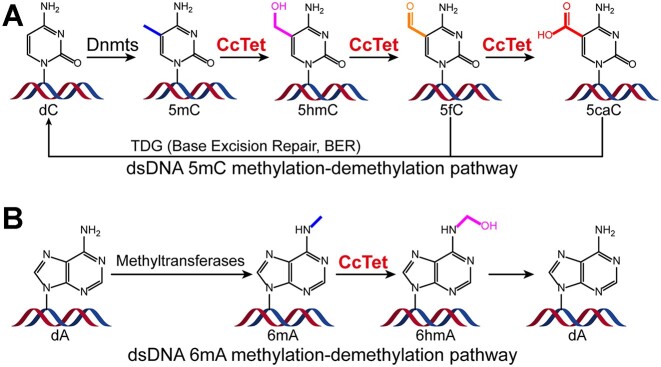
Schematic diagrams of dynamic eukaryotic dsDNA 5mC and 6mA methylation–demethylation pathways. Eukaryotic dsDNA (**A**) 5mC and (**B**) 6mA methylation–demethylation pathways. Members of the DNMT methyltransferase family methylate dsDNA to form 5mC; demethylation is mediated by a Tet dioxygenase (e.g. CcTet) and TDG. The methyltransferases methylate DNA to form 6mA; in *C. cinerea*, demethylation is catalyzed by the bifunctional DNA 5mC/6mA demethylase CcTet.

Both 5mC and 6mA modifications are generated through a relatively simple process: direct addition of a methyl group by methyltransferases (16,21,22). In contrast, the catalytic removal of these methyl groups is relatively complicated. 5mC active demethylation is catalyzed by a pair of enzymes in mammals (22). First, ten-eleven translocation dioxygenases (Tets) iteratively oxidize the methyl group of 5mC to hydroxymethyl group (5-hydroxymethylcytosine, 5hmC), formyl group (5-formylcytosine, 5fC) and carboxyl group (5-carboxylcytosine, 5caC) by utilizing the family conserved Fe(II)/2-oxoglutarate-dependent Jumonji C (JmjC) catalytic jelly-roll core and oxygen as the co-substrate (23–27) (Figure 1A). Subsequently, 5fC and 5caC are recognized and excised to apurinic/apyrimidinic sites by a thymine-DNA glycosylase (TDG), and then are further repaired back to unmethylated cytosine through the conventional mammalian base excision repair pathway (26,28), establishing the dynamic and closed cycle of mammalian DNA methylation–demethylation regulation pathway. So far, Tet homologs with conventional 5mC oxidation activity were reported in several species, including Tets from humans (hTet1, hTet2 and hTet3), unicellular amoeboflagellate *Naegleria gruberi* (NgTet1) and the mushroom *Coprinopsis cinerea* (CcTet) (26,27,29–32). Notably, a Tet homolog from the unicellular green alga *Chlamydomonas reinhardtii*, CMD1, transfers an l-ascorbate molecule to the methyl group of 5mC to form a novel C5-glyceryl-methylcytosine modification, regulating the photosynthesis for adaptation under intense light conditions (29,33) (Supplementary Figure S1). These findings suggest that Tet dioxygenases exhibit distinct species-specific catalytic functions despite their common evolutionary origins and highly conserved catalytic core.

To date, eukaryotic dsDNA 6mA demethylases have been less thoroughly characterized than 5mC demethylases. We recently demonstrated that CcTet, which was previously identified as a *C. cinerea* 5mC dioxygenase, also functions as a dsDNA 6mA demethylase (32,34). Its catalysis not only of conventional 5mC oxidation during the DNA active demethylation process, but also of dsDNA 6mA demethylation makes it a bifunctional dsDNA demethylase (Figure 1B). Kinetic studies indicate a slight preference (∼7-fold) of CcTet for 5mC over 6mA (*K*_cat_/*K*_m_= 2.64 mM^−1^ s^−1^ for catalyzing 5mC·G base; *K*_cat_/*K*_m_= 0.38 mM^−1^ s^−1^ for catalyzing 6mA·T base) (34). Further study on the 6mA demethylation mechanism suggested that a nonconserved residue CcTet Gly331 specifically facilitates 6mA recognition. The absence of a side chain on this residue vacates additional space for 6mA accommodation inside the active pocket. Contrarily, the corresponding residue in other Tets (Asn1387 in hTet2, Asp234 in NgTet1 and Asp350 in CMD1) utilizes a side chain to stabilize the *N*^4^ atom of 5mC pyrimidine ring through H-bond, and introduces direct clashes with the *N*^6^-methyl group of 6mA, leading to their substrate preference on 5mC over 6mA. Besides Gly331, another nonconserved residue CcTet Asp337 inside the active pocket also contributes to 6mA recognition through hydrophobic interactions. Unexpectedly, the mutagenesis of Asp337 to hydrophobic amino acids sharply reduces 5mC oxidation activity of CcTet, among which CcTet D337F mutation completely abolishes the 5mC oxidation activity without interfering that of 6mA demethylation, indicating a prominent distinct but unclear 5mC oxidation mechanism of CcTet compared to other conventional Tet family 5mC dioxygenases.

In the present study, we sought to elucidate the 5mC oxidation mechanism by representing the crystal structure of CcTet in complex with 5mC-containing 12-bp dsDNA oligo, as well as the distinct CcTet structural features that enable its bifunctionality. Our results suggested that CcTet recognizes 5mC-dsDNA substrate in the way that alternates to that of 6mA-dsDNA. Moreover, CcTet Asp337 plays an essential central role in superseding the loss of a critical H-bond in 5mC stabilization observed in the conventional Tets, facilitating its dual function in catalyzing both 5mC oxidation and 6mA demethylation on dsDNA. These findings facilitate an enhanced understanding of Tet enzyme function in the critical dsDNA 5mC and 6mA demethylation pathways. Furthermore, it provides valuable information for the discovery of small molecular probes to target Tets during DNA active demethylation processes ultimately.

## Materials and methods

### Molecular cloning and protein purification

The gene of CcTet-ΔN16 and its single site mutations were subcloned to the pET-SUMO vector with an N-terminal His-Sumo1 tag and expressed in *Escherichia coli* BL21(DE3), and the enzymes were purified according to our previous report (34). Briefly, the proteins were purified by using Ni-NTA affinity column. After the removal of the N-terminal tag by Ulp1 protease, the proteins were purified by using HiTrap QFF anion-exchange column followed by the GE HiLoad 16/600 Superdex75 prep grade gel-filtration column with running buffer containing 20 mM HEPES (pH 7.0), 100 mM NaCl and 1 mM DTT. The purified proteins were pooled and concentrated to 30 mg/ml for subsequent enzymatic studies and crystallization. The concentrations of the proteins were quantified by using Bio-Rad Bradford reagent.

### Crystallization of CcTet–5mC-dsDNA complex

The purified 30 mg/ml CcTet-ΔN16 was first incubated with 2 mM 2-oxoglutarate inactive analog *N*-oxalylglycine (NOG), 1 mM MnCl_2_ and 1:1.2 molar ratio 12-bp dsDNA oligo containing a single 5mC modification (sequences: 5′-CGATC|5mC|GCTACG-3′; 5′-CGTAGCTGATCG-3′) for 1 h under 4°C. Subsequently, it was mixed with equal volume of reservoir solution and equilibrated with 100 μl of the reservoir solution under 277 K for 1 month. Plate-shaped crystals were observed in the reservoir solution containing 20 mM sodium formate and 10% PEG8000. The crystals were subsequently soaked in protectant containing mother liquor with 30% glycerol (v/v) and flash frozen in liquid nitrogen for X-ray diffraction.

### Data collection and structure determination

The diffraction data from a single crystal were collected at BL19U1 beamline of National Facility for Protein Science in Shanghai at Shanghai Synchrotron Radiation Facility, equipped with an CMOS hybrid pixel Pilatus3 6M detector using X-rays tuned to wavelength of 0.9875 Å. The diffraction data were further integrated and scaled with space group *P*2_1_2_1_2_1_ by using autoPROC software (Supplementary Table S1) (35). The structure was subsequently determined by molecular replacement using PHASER with reported human Tet2 structure (PDB code: 4NM6) as the search model (26,36). The structure was subsequently refined by using Phenix-refine and the model was conducted by graphics program Coot (37,38). The protein structure viewer was performed by PyMol software.

### Enzymatic 5mC demethylation assays

The enzymatic assays were performed according to the previous report (34). Briefly, 1 μM CcTet-ΔN16 enzymes were mixed with 1 μM of 19-bp single 5mC-containing dsDNA (DNA sequences: 5′-TCTGGAA(5mC)GGAATTCTTCA-3′; 5′-TGAAGAATTCCGTTCCAGA-3′) in the buffer containing 50 mM HEPES, pH 7.0, 100 mM NaCl, 400 μM α-ketoglutarate and 200 μM Fe(NH_4_)_2_(SO_4_)_2_. The reactions were incubated for 30 min at 37°C in triplicates. The reactions were quenched by heating to 95°C for 5 min and immediately cooled in an ice bath. Subsequently, the products were digested with Nuclease P1 (catalog no. M0660S; New England Biolabs) and Calf Intestinal Alkaline Phosphatase (catalog no. 18009019; Invitrogen) at 37°C. The final solution was monitored by quantitative mass spectrometry (LC–MS/MS).

### LC–MS/MS detection and analysis

The nucleosides were separated by an SB-Aq C18 column (2.1 mm × 100 mm, 1.8 μm; Agilent Technologies) and detected by ABSCIEX QTRAP 6500 systems in positive ion mode. The quantitative analysis was performed according to the previous published methods (34).

### Microscale thermophoresis binding assays

The assay was performed with a NanoTemper Monolith NT.115 instrument. Ten micromolar CcTet-ΔN16 or its mutants were labeled with fluorescence dye NT-495 according to manufacturer’s protocol. Ten microliters of labeled protein was subsequently mixed with various concentrations of 19-bp 5mC-dsDNA oligo and incubated at room temperature for 5 min. For measurements, samples were filled into capillaries and the assay was conducted on the NanoTemper Monolith NT.115 instrument (NanoTemper Technologies) with 50–80% excitation power and 40% microscale thermophoresis (MST) power. Statistical analysis was performed using NanoTemper analysis software.

## Results and discussion

### Overall structure of the CcTet–5mC-dsDNA complex

To investigate the 5mC oxidation mechanism of CcTet, a version of the enzyme was subcloned and purified with the 16 amino acids at the N-terminal deleted (CcTet-Δ16). The complex of CcTet-Δ16 and 12-bp 5mC-dsDNA oligo was crystallized at 277 K in the presence of manganese(II) (Mn^2+^) and a catalytically inactive analog of 2-oxoglutarate, NOG. After incubation of ∼1 month, plate-shaped crystals were observed and flash frozen in liquid nitrogen. Diffraction data were scaled to 2.3Å resolution with the *P*2_1_2_1_2_1_ space group (Supplementary Table S1). The resolved structure suggested that there were four CcTet–5mC-dsDNA complexes in each asymmetric unit (Supplementary Figures S2 and S3). CcTet molecules from each of the complexes interacted with each other predominantly through interactions between the α3 helix and the β1 strand, which were located on the opposite side of the enzyme from the active pocket entrance and the DNA binding site, respectively. Moreover, the dsDNA oligos from each of the complexes formed a head-to-tail linear packing pattern, further stabilizing complex packing within the crystals.

**Figure 2.**
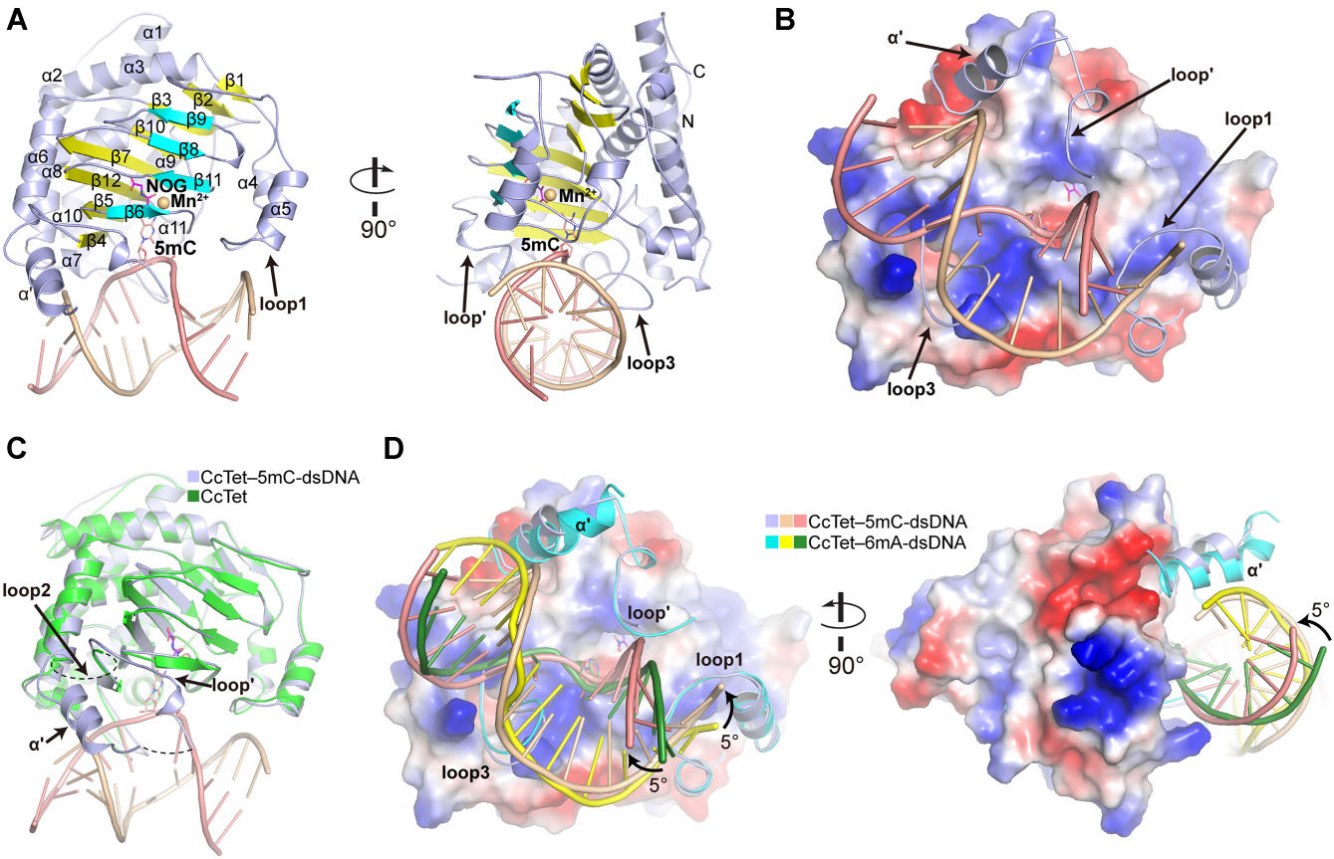
Crystal structure of CcTet in complex with a 12-bp 5mC-containing dsDNA oligo. (**A**) Crystal structure of the CcTet**–**5mC-dsDNA complex. NOG, 5mC and Mn^2+^ are shown in stick or sphere form, respectively. CcTet secondary structures and functional loop regions are labeled. (**B**) Electrostatic surface map of CcTet with functional loop regions shown and labeled. (**C**) Superposition of the CcTet**–**5mC-dsDNA complex with the apo CcTet structure. (**D**) Superposition of the CcTet**–**5mC-dsDNA complex with the CcTet**–**6mA-dsDNA complex. Arrows indicate the direction of 5mC-dsDNA rotation compared to 6mA-dsDNA upon CcTet binding.

In complex with dsDNA, the middle of the enzyme contained two anti-parallel β-sheet layers comprising 12 β-strands total, with one sheet containing β6-β11-β8-β9 and the other containing β4-β5-β12-β7-β10-β3-β2-β1 (Figure 2A). These sheets formed a jelly-roll catalytic pocket to accommodate cofactors and substrates. An N-terminal helix bundle domain (α1, α2, α3, α6, α7 and α8) attached opposite of the active pocket entrance to stabilize the jelly-roll core. Notably, the corresponding α-helical bundle in hTet2 is stabilized by two zinc(II) ions that are not required in CcTet or NgTet1 (26,27,30,34). The dsDNA oligo was bound to CcTet at the positively charged area around the active pocket entrance, and was predominantly stabilized by three CcTet loop regions: N-terminal loop1 (residues 79–108, α4-loop-α5), α′/loop′ (residues 163–210, originating from the flexible loop2) and loop3 (residues 228–240) (Figure 2B). Loop1 and loop3 inserted into the dsDNA minor groove, whereas the α′/loop′ region bound to the edges of the dsDNA strands near the major groove.

The dsDNA-bound CcTet adopted a similar conformation to the apo form CcTet (Protein Data Bank, PDB code 7VPN) with the average root-mean-square deviation (RMSD) value of 0.360. A major difference between the two structures was that the flexible and structural disordered loop2 region in apo form CcTet structure was partially restructured to a new α-helix (α′) and loop region (loop′) upon dsDNA’s binding (Figure 2C). Superposition of CcTet–5mC-dsDNA with the reported structure of CcTet–6mA-dsDNA complex (PDB code 7W5P) suggested a high overall similarity (average RMSD value 0.424). Unexpectedly, the dsDNA strands in the CcTet–5mC-dsDNA complex were rotated ∼5° anticlockwise due to compression by the loop1 and α′/loop′ regions; this indicated an alternative mode of 5mC-dsDNA binding compared to 6mA-dsDNA (Figure 2D) (34).

**Figure 3.**
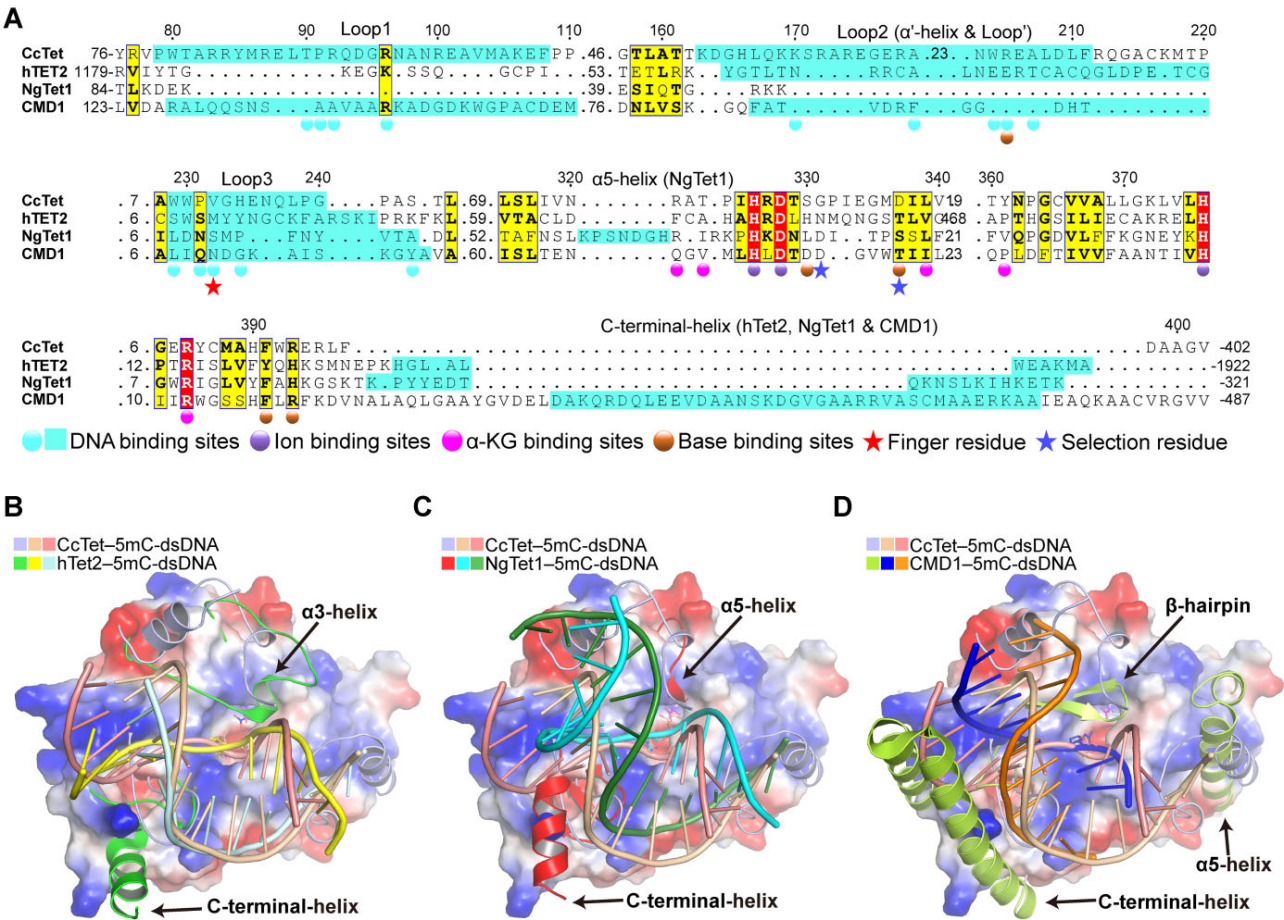
Structural comparison of the CcTet–5mC-dsDNA, hTet2–5mC-dsDNA, NgTet1–5mC-dsDNA and CMD1–5mC-dsDNA complexes. (**A**) Structural alignment of CcTet, hTet1, NgTet1 and CMD1. Residues involved in NOG/Mn^2+^/dsDNA binding are labeled with spheres. Superposition of CcTet–5mC-dsDNA with (**B**) hTet2–5mC-dsDNA, (**C**) NgTet1–5mC-dsDNA and (**D**) CMD1–5mC-dsDNA. Arrows indicate nonconserved secondary structural regions involved in dsDNA binding.

CcTet–5mC-dsDNA was compared with other reported Tet–dsDNA complexes such as hTet2–5mC-dsDNA (PDB code 4NM6), NgTet1–5mC-dsDNA (PDB code 4LT5) and CMD1–5mC-dsDNA (PDB code 7CY6) complexes via structure-based sequence alignment and superposition (Figure 3A). The analysis suggested that the three loop regions of CcTet were not conserved among Tet homologs. CcTet lacked the key C-terminal α-helix region that mediates dsDNA binding in other Tets (Figure 3B–D). Instead, CcTet loop1 region was inserted into the dsDNA minor groove, contributing to dsDNA stabilization and compensating for the absence of the C-terminal α-helix region. Moreover, all of the examined Tet homologs required a region corresponding to the CcTet loop3 region, in which the key finger residue that facilitated substrate base flipping was located. In contrast, the amino acid sequences and secondary structures corresponding to the CcTet α′/loop′ region varied greatly between enzymes. NgTet1 lacked such a region for dsDNA binding, indicating a lesser role of this region in dsDNA recognition and stabilization. Our previous study demonstrated that CcTet has weak activity on single-stranded (ss) DNA as do NgTet1 and hTet2 (32). Structural analysis here suggested that these enzymes did not form secondary structures that prohibited ssDNA binding, explaining their weak ssDNA catalytic activity. Overall, these observations indicated that CcTet had a distinct mechanism of 5mC-dsDNA substrate recognition compared to other Tets.

### dsDNA substrate recognition

**Figure 4.**
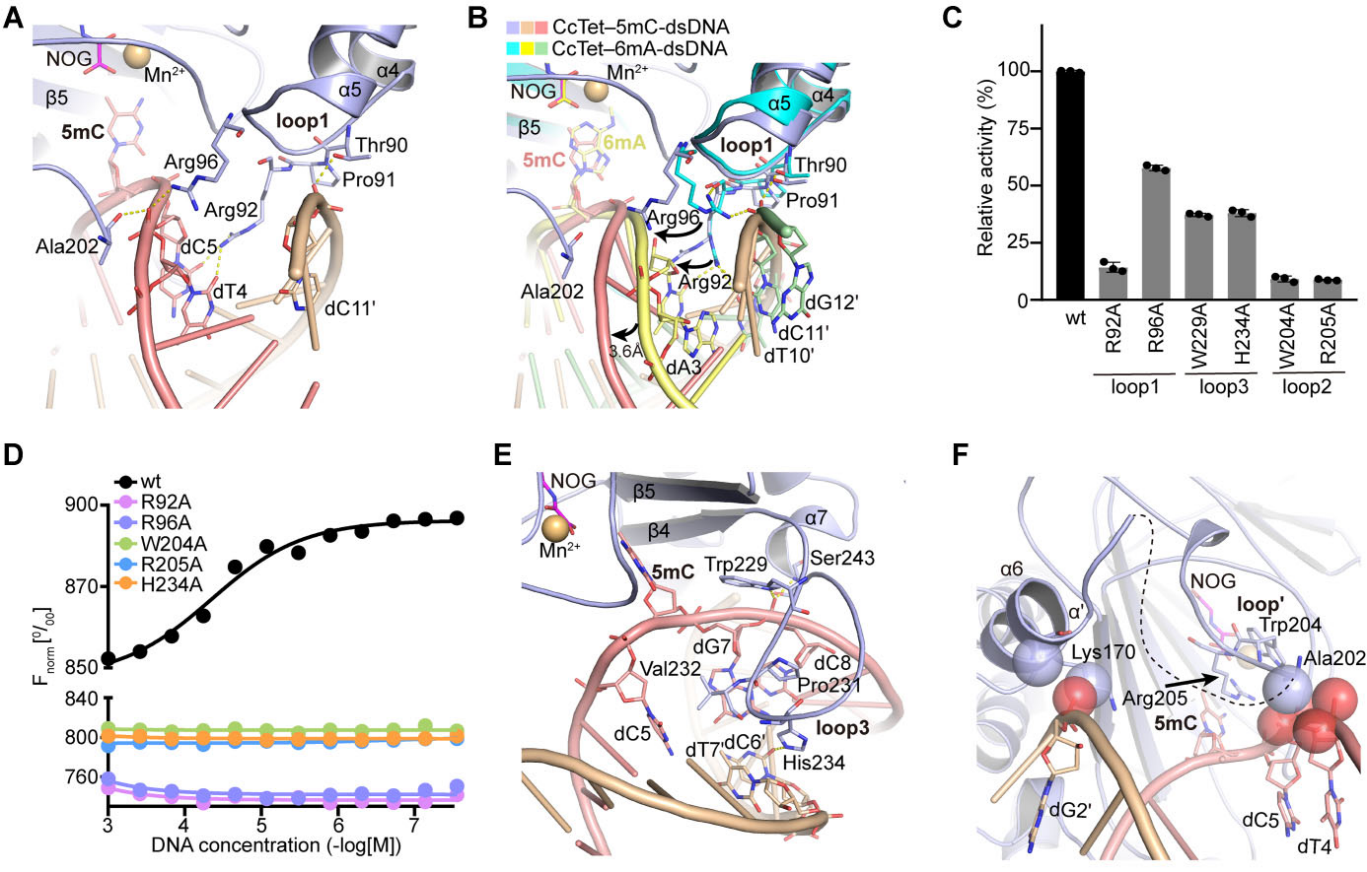
CcTet DNA recognition. (**A**) DNA binding by the CcTet loop1 region. CcTet residues involved in dsDNA interactions are shown in stick form and labeled. (**B**) Superposition of the loop1 region in the CcTet–5mC-dsDNA and CcTet–6mA-dsDNA complexes. Arrows indicate residue side chain or dsDNA backbone movements in the CcTet–5mC-dsDNA compared to the CcTet–6mA-dsDNA complex. (**C**) Mutagenized CcTet 5mA-dsDNA demethylation activity. *n* = 3 biologically independent experiments. Data are shown as the mean ± standard deviation. (**D**) MST binding affinity of mutagenized CcTet for the 5mC-dsDNA oligo. (**E**) DNA binding by the CcTet loop3 region. (**F**) DNA binding by the CcTet loop2 region. CcTet atoms involved in dsDNA binding are shown in sphere form.

The loop1 region of dsDNA-bound CcTet was a characteristic structural feature for substrate stabilization that was absent in the other Tets (Figure 4A). The side chains of Thr90 and Pro91, which were located on loop1, formed hydrophilic interactions with the edge of the dsDNA strand that did not contain the 5mC substrate. The Thr90 side chain also hydrophobically interacted with the backbone phosphate of dC11′, further stabilizing the dsDNA. Remarkably, compared to 6mA-dsDNA-bound CcTet, the Arg92 and Arg96 side chains swung ∼80° and ∼110°, respectively, toward the dsDNA strand containing the 5mC substrate. This eliminated the hydrophilic interactions observed between these residues and the dT10′, dC11′ and dG12′ located on the complementary strand in the CcTet–6mA-dsDNA complex (Figure 4B). Instead, Arg92 and Arg96 formed salt bridge interactions with the *O*^2^ atoms of dT4 and dC5, or with the backbone phosphate of dC5, pushing the dsDNA strands outward by ∼3.6 Å compared to the 6mA-dsDNA bound form. This demonstrated the alternative CcTet binding mechanism of 5mC-dsDNA compared to 6mA-dsDNA. Mutagenesis of Arg92 and Arg96 to alanine residues abolished the binding of dsDNA on CcTet, and reduced the enzymatic activity of CcTet in catalyzing 5mC oxidation more drastic than that of 6mA (Figure 4C and D). These observations suggested that the characteristic loop1 region of CcTet played a key role in recognition and catalysis of 5mC-containing dsDNA.

In contrast to the distinctive loop1 region present in CcTet, a structurally conserved loop region corresponding to CcTet loop3 was shared by all of the examined Tets (Figure 3B–D). It was inserted into the minor groove of the dsDNA substrate, enabling the key finger residue within the loop to push the substrate into the active pocket (Figure 4E). Specifically, in the CcTet–5mC-dsDNA complex, the finger residue Val232 was stacked between dC5 and dG7 through hydrophobic interactions; the stacked residue then pushed the 5mC base, causing it to flip into the active pocket for subsequent catalysis. The side chains of Trp229 and Pro231 (which were also located in loop3) stacked the DNA strand containing 5mC through hydrophobic interactions, and Ser243 side chain formed hydrophilic interactions with the backbone phosphate of dC8, stabilizing the DNA for further catalysis. Notably, the W229A mutant decreases the enzymatic activity of 5mC oxidation much more than that of 6mA oxidation, which may be due to the alternative binding manner of 5mC/6mA-containing dsDNA on CcTet (Figure 2D). The His234 side chain also moved ∼2.6 Å toward the complementary DNA strand compared to its location in the CcTet–6mA-dsDNA complex, forming an H-bond with the *O*^2^ atom of dC6′ (Supplementary Figure 4A). Mutagenesis of His234 to alanine abolished dsDNA binding to CcTet and sharply reduced enzymatic activity, indicating a key role of this residue in dsDNA binding (Figure 4C and D, and Supplementary Table S2).

Besides loop1 and loop3, the structural disordered loop2 observed in apo form CcTet structure was partially restructured to form the α′-helix and loop′ upon dsDNA’s binding (Figure 4F). In complex with 5mC-dsDNA, the α′-helix was ∼8 Å closer to the complementary strand than it was in complex with 6mA-dsDNA. This change pushed the DNA strand ∼5 Å closer to the active pocket through hydrophobic interactions between the Lys170 side chain (located on the α′-helix) and the backbone phosphate of dG2′ (Supplementary Figure S4B). Additionally, Ala202 side chain (located on the loop′) formed hydrophobic interactions with dT4 and dC5 backbones. Moreover, Arg205 (located in the loop) was stabilized via hydrophobic interactions with Trp204 side chain, and contributed significantly to 5mC base stabilization inside the active pocket. Similar to the observations on Arg92 and Arg96, the stabilization of 5mC-dsDNA requires Trp204 and Arg205 to contribute more due to the contortion of the 5mC-dsDNA on CcTet compared to that of 6mA-dsDNA, and thereby the mutagenesis of Trp204 and Arg205 to alanine affects 5mC oxidation much more than that of 6mA oxidation (Figure 4C and D). Notably, the regions corresponding to the CcTet α′/loop′ in hTet2–dsDNA and NgTet1–dsDNA complex structures are short α-helices (α3- and α5-helices in hTet2 and NgTet1, respectively); in CMD1, a β-hairpin forms upon dsDNA binding, which contributes weak, transient hydrophobic interactions with the dsDNA (Figure 3B–D). This strongly suggests that the disordered CcTet loop2 underwent structural reorganization during dsDNA binding to further contribute to 5mC/6mA-dsDNA recognition and stabilization. These structural features demonstrated a conventional but distinct mechanism of tertiary structural 5mC-dsDNA recognition via the secondary structures of CcTet loop1, loop2 (α′/loop′) and loop3.

### Cofactors’ recognition

**Figure 5.**
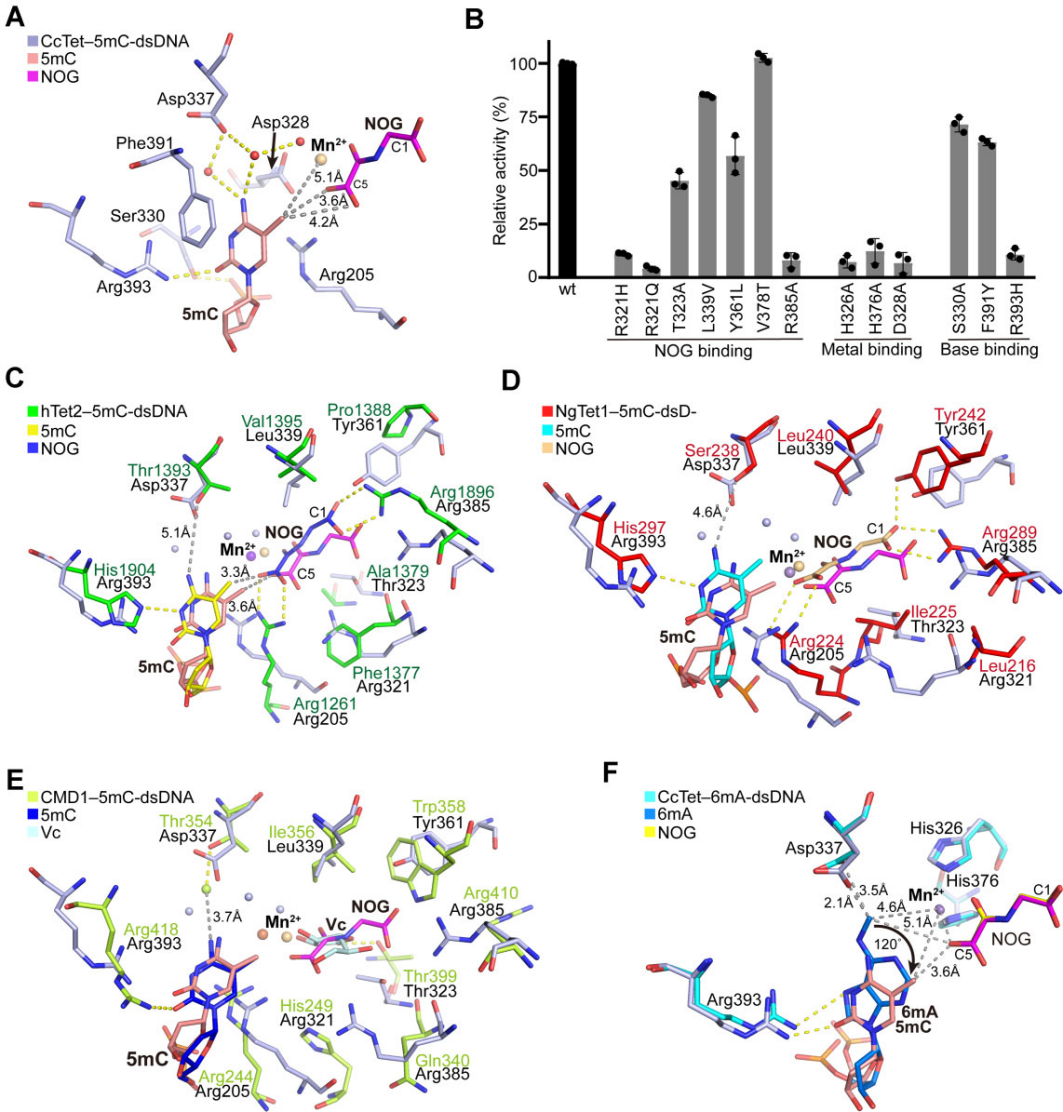
CcTet recognition of the 5mC substrate. (**A**) Interactions between CcTet and a 5mC base. Residues inside the active pocket that are involved in 5mC recognition are shown in stick form and labeled. (**B**) Mutagenized CcTet 5mA-dsDNA demethylation activity. *n* = 3 biologically independent experiments. Data are presented as the mean ± standard deviation. Superposition of the CcTet active pocket with those of (**C**) hTet2, (**D**) NgTet1 and (**E**) CMD1. (**F**) Superposition of the CcTet active pockets in the CcTet–5mC-dsDNA and CcTet–6mA-dsDNA complexes. Arrows indicate the direction of 5mC rotation compared to 6mA.

The active pocket of CcTet predominantly consisted of residues from the two anti-parallel β-sheet layers in the jelly-roll catalytic core, and most of these residues were conserved among the Tet family enzymes (Figure 3A). The cofactors Mn^2+^ and NOG and the flipped 5mC substrate were stabilized in the correct positions and orientations inside the active pocket through multiple hydrophobic and hydrophilic interactions (Figure 5A and Supplementary Figure S5). Mn^2+^ was primarily stabilized via octahedral coordinate interactions with the side chains of His326, His376 and Asp328, a water molecule, and the NOG *C*^5^-carboxyl and *C*^4^-ketone groups. Structural sequence alignment suggested that these three residues were highly conserved among all of the examined Tet and AlkB enzymes (Figure 3A). Mutagenesis of any of these residues to alanine eliminated CcTet 5mC oxidation activity, indicating a common oxidation mechanism among superfamily members (Figure 5B and Supplementary Table S2).

NOG was stabilized through hydrophilic and hydrophobic interactions with several nearby residues, which were less conserved among Tet superfamily members (Figure 3A). The *C*^1^ carboxyl group of NOG interacts hydrophilically with Arg385, Thr323 and Tyr361 side chains, among which Arg385 was highly conserved throughout the superfamily and played a critical role in NOG stabilization by forming salt bridges with the *C*^1^ carboxyl group (Figures 3A and 5A). Mutagenesis of CcTet Arg385 to alanine fully abolished enzymatic activity (Figure 5B). Furthermore, the Thr323 and Tyr361 side chains formed H-bonds with the NOG *C*^1^ carboxyl group. Tyr361 and Thr323 were less conserved among superfamily members than Arg385, but the corresponding residues in other enzymes were consistently hydrophobic (alanine/isoleucine/valine for Thr323; valine/proline for Tyr361). Mutagenesis of Thr323 to alanine (T323A) or Tyr361 to leucine (Y361L) decreases the enzymatic activity of CcTet, indicating the critical role of the hydrophilic electric charge environment around this spot. Notably, the residue corresponding to CcTet Thr323 was Thr399 in CMD1, which provided the only hydrophilic interaction stabilizing the cofactor (substrate) l-ascorbate. Further enzymatic activity assay suggested that the presence of 2-oxoglutarate is required for the enzymatic activity of CcTet, and CcTet shows little enzymatic activity on ascorbate in the absence of 2-oxoglutarate, indicating a distinct catalytic mechanism between CcTet and CMD1 (Supplementary Figure S6).

In contrast to the numerous interactions with the NOG *C*^1^ carboxyl group, there were few interactions observed at the *C*^5^ carboxyl group. The *C*^5^ carboxyl participated in coordinate interactions with the metal ion and weak hydrophilic interactions with the highly conserved active residues His326 and Asp328, in addition to forming an H-bond with the side chain of the nonconserved residue Arg321 (Figure 5A and E). Sequence and structural alignments suggested that the residues corresponding to CcTet Arg321 (Phe1377 in hTet2, Leu216 in NgTet1 and Gln340 in CMD1) had short side chains or were hydrophobic; CcTet Arg321 mutagenesis to glutamine or another positively charged residue nearly eliminated enzymatic activity. This suggested a distinct and important role of Arg321 in CcTet 5mC oxidation (Figure 5B). In addition to hydrophilic interactions, NOG was further stabilized through hydrophobic interactions with the Leu339, Thr323 and Val378 side chains. Mutagenesis of CcTet Leu339 to valine (L339V) and Leu378 to threonine (L378T) (the corresponding residues in hTet2) only slightly decreased CcTet 5mC oxidation activity. These observations suggested that CcTet employed a NOG recognition mechanism that was conserved within the Tet superfamily.

### 5mC recognition and catalysis

In the CcTet active pocket, the 5mC base was stabilized through multiple hydrophobic and hydrophilic interactions with surrounding highly conserved residues (Figures 3A and 5A and C–E). Specifically, the 5mC base was stacked between Phe391 and Arg205, which were conserved among the family members, with the exception of a Tyr (Tyr1902) in place of the Phe in hTet2. However, F391Y mutagenesis did not significantly interfere with CcTet activity, indicating that the hydrophobic ring, rather than the hydroxyl group of the side chain, predominantly contributed to 5mC base stabilization (Figure 5B). In contrast, although CcTet Arg205 was located on a unique secondary element (the nonconserved loop2) compared to the corresponding residues in hTet2 and NgTet1 (a short α-helix), mutagenesis of CcTet Arg205 to alanine abolished CcTet activity, confirming that the disordered loop2 of CcTet contributed significantly to the specific and distinct recognition of dsDNA and 5mC bases in CcTet.

In addition to the stacking π–π interactions, the *O*^2^ atom of the 5mC base formed an H-bond with the Arg393 side chain. Sequence alignment suggested that the corresponding residues in hTet2 and NgTet1 were His1904 and His297, respectively (Figure 3A). Although Arg and His residues contributed similarly to base stabilization via an H-bond, R393H mutagenesis in CcTet drastically reduced enzyme activity, indicating that CcTet achieved 5mC recognition and oxidation through a mechanism that differed from those of other Tet members (Figure 5B). In addition to base recognition, the CcTet Ser330 side chain hydrophilically interacted with the 5mC phosphate group, further stabilizing the substrate in the correct orientation. S330A mutagenesis only slightly reduced CcTet activity, suggesting a subordinate role of this residue in catalysis.

Superposition of the active pockets of the CcTet–5mC-dsDNA and CcTet–6mA-dsDNA complexes revealed a similar base recognition mechanism (Figure 5F). The 5mC base was rotated ∼120° clockwise inside the active pocket compared to the 6mA base. The *C*^5^ methyl group was significantly closer to the NOG *C*^5^ carboxyl group than the 6mA *N*^6^ methyl group (3.6 and 5.1 Å, respectively), and was also slightly further away from the Mn^2+^ ion (5.1 Å) than the 6mA *N*^6^ methyl group (4.6 Å). Remarkably, previous study revealed that CcTet stabilized the 6mA base via hydrophobic interactions between the Asp337 side chain and the 6mA *N*^6^-methyl group. In contrast, the Asp337 side chain rotated ∼90° in complex with 5mC-dsDNA to form two indirect hydrophilic interactions (through two water molecules) with the *N*^4^ atom of the 5mC pyrimidine ring, further stabilizing the 5mC base inside the active pocket (Figure 5A).

**Figure 6.**
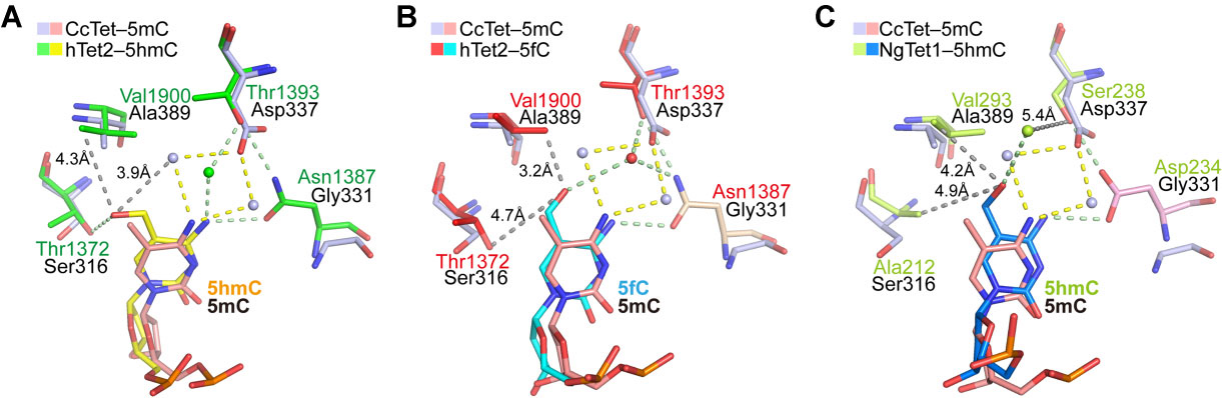
Superposition of active pockets in the CcTet–5mC-dsDNA, hTet2–5hmC-dsDNA, hTet2–5fC-dsDNA and NgTet1–5hmC-dsDNA complexes. Superposition of the CcTet–5mC-dsDNA complex active pocket with that of (**A**) hTet2–5hmC-dsDNA, (**B**) hTet2–5fC-dsDNA and (**C**) NgTet1**–**5hmC-dsDNA.

Further sequence alignment and structural superposition of CcTet–5mC-dsDNA with other Tet–dsDNA complexes suggested a distinct 5mC recognition mechanism. Each conventional Tet (hTet2, NgTet1 and CMD1) utilized a key residue corresponding to CcTet Gly337 (Thr1393, Ser238 and Thr354, respectively) to stabilize 5mC via a single weak indirect hydrophilic interaction between the side chain and the 5mC *N*^4^ atom of 5mC; each conventional Tet also contained another key residue corresponding to CcTet Gly331 (Asn1387, Asp234 and Asp350, respectively) that formed an additional direct H-bond with the 5mC *N*^4^ atom to further stabilize 5mC and block 6mA recognition (Figures 3A, 5C–E and 6A and B) (34).

The presence of Gly331 in CcTet vacated space occupied by the side chains of the corresponding residues in the other Tets, allowing accommodation of the 6mA *N*^6^ methyl group. However, this eliminated the critical H-bond between the 5mC *N*^4^ atom and Asn1387/Asp234/Asp350, which stabilized 5mC in the other enzymes. Thus, 5mC stabilization was weaker in the CcTet active pocket compared to the other Tets. To compensate for the loss of this H-bond and to strengthen 5mC stabilization, the nonconserved CcTet residue Asp337 formed two indirect hydrophilic interactions with the 5mC *N*^4^ atom. In hTet2, N1387G mutagenesis generated a novel capacity of the enzyme to catalyze 6mA demethylation. However, mutagenesis of the CcTet residue Asp337 to Thr, Ser or a hydrophobic residue eliminated 5mC oxidative activity, suggesting an essential and central role of CcTet Asp337 in stabilizing 5mC that superseded the roles of residues corresponding to Gly331 in other Tet enzymes (Figure 5B) (34). These observations indicated that CcTet employed a distinct 5mC oxidative mechanism compared to other Tets, and explained our previous observations that CcTet Asp337 mutagenesis to a hydrophobic residue abolished 5mC oxidative activity.

Superposition of the CcTet–5mC-dsDNA complex with hTet2–5hmC/5fC-dsDNA (PDB codes 5DEU and 5D9Y) and NgTet1–5hmC-dsDNA (PDB code 5CG8) suggested that Asp337 may also have contributed to stabilization of 5mC oxidation intermediates (5hmC and 5fC) and even of the final oxidation product (5caC) via direct H-bonds with the *C*^5^ hydroxyl, formyl or carboxyl groups (Figure 6). hTet2 and NgTet1 utilized key residues corresponding to CcTet Asp337 (Thr1393 and Ser238, respectively) to stabilize the *C*^5^ formyl group of 5fC or the *C*^5^ hydroxyl group of 5hmC through a hydrophilic interaction using a single water molecule, similar to the mechanism of 5mC stabilization. Because the CcTet Asp337 side chain was longer than the side chains of the corresponding residues in other Tets, it most likely formed a direct H-bond with the *C*^5^ hydroxyl, formyl or carboxyl group, stabilizing the substrate in the correct location within the active pocket for further catalysis (34). In contrast, the 5hmC *C*^5^ hydroxyl group was rotated by ∼180° in complex with hTet2 compared to NgTet1, pointing toward hTet2 Thr1372. CcTet Aps337 may have facilitated flipping of this group during oxidation to the formyl intermediate and stabilized the final oxidation product 5caC (27).

## Conclusions

The eukaryotic epigenetic dsDNA modifications 5mC and 6mA have indispensable regulatory roles. We previously identified an atypical bifunctional dsDNA 5mC/6mA demethylase, CcTet, in *C. cinerea*. A mechanistic study of CcTet 6mA-dsDNA catalysis previously suggested that the nonconserved CcTet residues Gly331 and Asp337 facilitated 6mA recognition and catalysis. In the present study, we elucidated the molecular mechanism by which CcTet catalyzed 5mC oxidation in the 5mC-dsDNA active demethylation pathway. The CcTet residue Asp337 was found to play a central role in 5mC stabilization. Asp337 mutagenesis to a hydrophobic residue (e.g. Phe) maintained 6mA activity, leading to formation of similar hydrophobic interactions with the 6mA *N*^6^-methyl group, but abolished 5mC activity by eliminating the H-bond with 5mC *N*^4^. Moreover, residues corresponding to CcTet Gly331 in conventional Tets stabilized 5mC, 5hmC and 5fC bases through an H-bond with the *N*^4^ atom of the 5mC pyrimidine ring, the 5hmC hydroxymethyl group or the 5fC formyl group. This indicated a distinct 5mC oxidation mechanism of CcTet compared to conventional Tets. These findings promote our understanding of Tet dioxygenase functions in the DNA demethylation pathway, and may facilitate the chemical probe discovery against Tet enzymes.

## Supplementary Material

gkae066_Supplemental_File

## Data Availability

The atomic coordinate and structure factor of the crystal structure were deposited to the Protein Data Bank (www.rcsb.org) under accession code 8JKK.
