# Supplementary material for: Molecular basis of an atypical dsDNA 5mC/6mA bifunctional dioxygenase CcTet from Coprinopsis cinerea in catalyzing dsDNA 5mC demethylation
Source: Nucleic Acids Res. 2024 Feb 7;52(7):3886–95. doi: 10.1093/nar/gkae066 (PMC11040006; doi:10.1093/nar/gkae066)
Supplement: gkae066_Supplemental_File [file gkae066_supplemental_file.doc]

Supplementary Information for

**Molecular basis of an atypical dsDNA 5mC/6mA bifunctional dioxygenase CcTet from *Coprinopsis cinerea* in catalyzing dsDNA 5mC demethylation**

Lin Zhang^1,§^, Yajuan Mu^1,§^, Tingting Li^1,§^, Jingyan Hu^1^, Houwen Lin^2,3^, Liang Zhang^1,*^

^1^Department of Pharmacology and Chemical Biology, State Key Laboratory of Systems Medicine for Cancer, Shanghai Jiao Tong University School of Medicine, Shanghai, 200025, China; ^2^Research Centre for Marine Drugs, State Key Laboratory of Oncogene and Related Genes, Department of Pharmacy, Ren Ji Hospital, School of Medicine, Shanghai Jiao Tong University, Shanghai 200127, China; ^3^Institute of Marine Biomedicine, Shenzhen Polytechnic, Shenzhen, 518055, China.

^§^ These authors contribute equally to this work.

*Correspondences: Liang Zhang, E-mail: [liangzhang2014@sjtu.edu.cn](mailto:liangzhang2014@sjtu.edu.cn)

This PDF file includes:

Supplementary Tables 1-2;

Supplementary Figures 1 to 6.

**Supplementary Table 1.** Data collection and refinement statistics.

|  | CcTet-Δ16–5mC-dsDNA |
| --- | --- |
| **Data collection** | |
| Space group | P2_1_2_1_2_1_ |
| Cell dimensions | |
| *a, b, c*(Å) | 86.569, 127.631, 212.236 |
| α, β, γ (°) | 90.00, 90.00, 90.00 |
| Wavelength (Å) | 0.9875 |
| Resolution (Å)* | 50.0-2.30 (2.38-2.30) |
| *R*_merge_ (%) | 16.5(109.6) |
| *I*/σ*I* | 14.9(2.6) |
| Completeness (%) | 99.7(99.8) |
| Redundancy | 13.4(13.6) |
| CC1/2 (%) | 99.5(95.5) |
|  | |
| **Refinement** | |
| Resolution (Å) | 50.0-2.3 |
| No.reflections | 94917 |
| *R*_work_/*R*_free_ | 0.19/0.22 |
| No.atoms | |
| Protein | 11916 |
| DNA | 1945 |
| Water | 936 |
| Ligand/ion | 44 |
|  | |
| B-factors | |
| Protein | 39.2 |
| DNA | 53.7 |
| Water | 38.8 |
| Ligand/ion | 29.7 |
| R.m.s deviations | |
| Bond lengths (Å) | 0.02 |
| Bond angles (°) | 1.63 |
| *Highest-resolution shell is shown in parentheses. | |

**Supplementary Table 2.** Enzymatic activities of CcTet mutants in catalyzing 5mC/6mA containing dsDNA substrates.

| CcTet mutants | Potential function | Activity on 5mC-dsDNA | Activity on 6mA-dsDNA |
| --- | --- | --- | --- |
| I318N | a-KG binding | Decreased | Decreased |
| R321H/Q |  | Decreased | Decreased |
| T323A/I |  | Decreased | Decreased |
| L339V |  | Slightly decreased | Maintained |
| Y361L |  | Decreased | Decreased |
| V378T |  | Maintained | Maintained |
| R385A |  | Decreased | Decreased |
| C387S/N |  | Maintained | Maintained |
| H326A | Metal binding | Abolished | Abolished |
| D328A |  | Abolished | Abolished |
| H376A |  | Abolished | Abolished |
| R92A | DNA binding (Loop1) | Decreased | Slightly decreased |
| R96A |  | Decreased | Slightly decreased |
| W204A | DNA binding (Loop2) | Decreased | Decreased |
| R205A |  | Decreased | Decreased |
| W229A | DNA binding (Loop3) | Decreased | Slightly decreased |
| V232G |  | Decreased | Decreased |
| H234A |  | Decreased | Decreased |
| S330A | Base binding | Decreased | Slightly Decreased |
| D337/T/S/**A**/**F** |  | Decreased/**Abolished** | **Maintained** |
| F391Y/A |  | Maintained/Decreased | Maintained/Decreased |
| R393H/A |  | Decreased | Slightly Decreased |


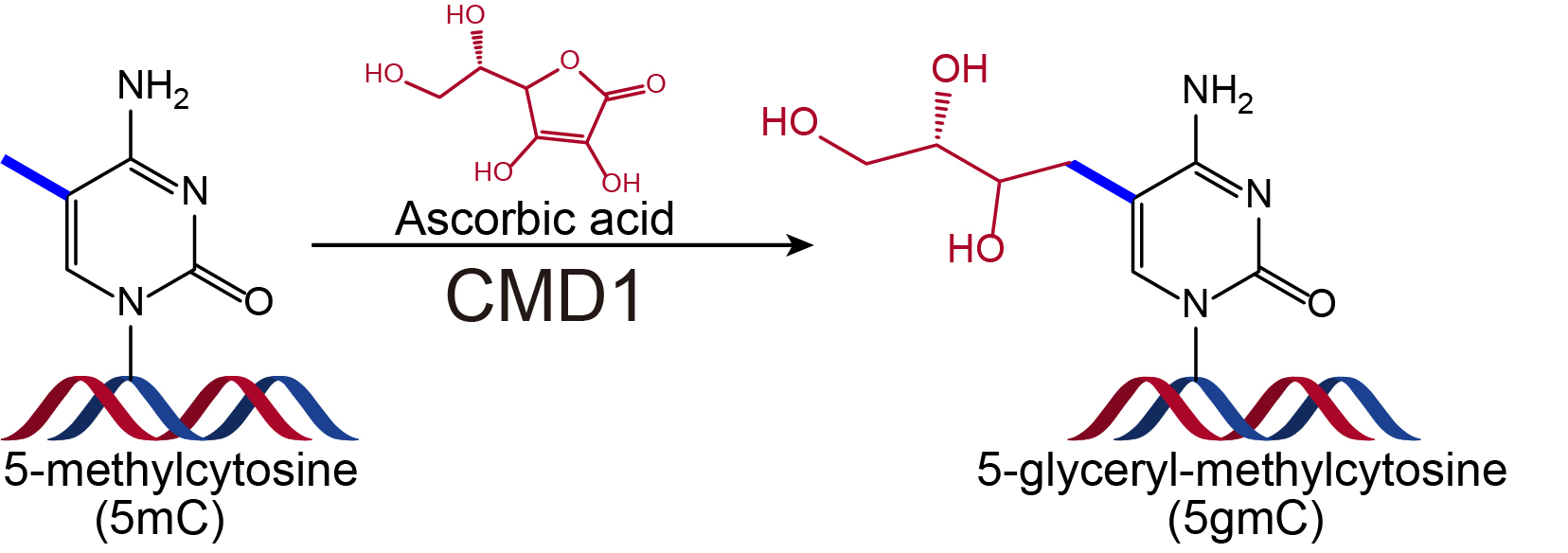


**Supplementary Figure 1.** Schematic diagrams of Tet dioxygenase family member CMD1 from *Chlamydomonas reinhardtii* in catalyzing L-ascorbic acid modification on 5mC-dsDNA.


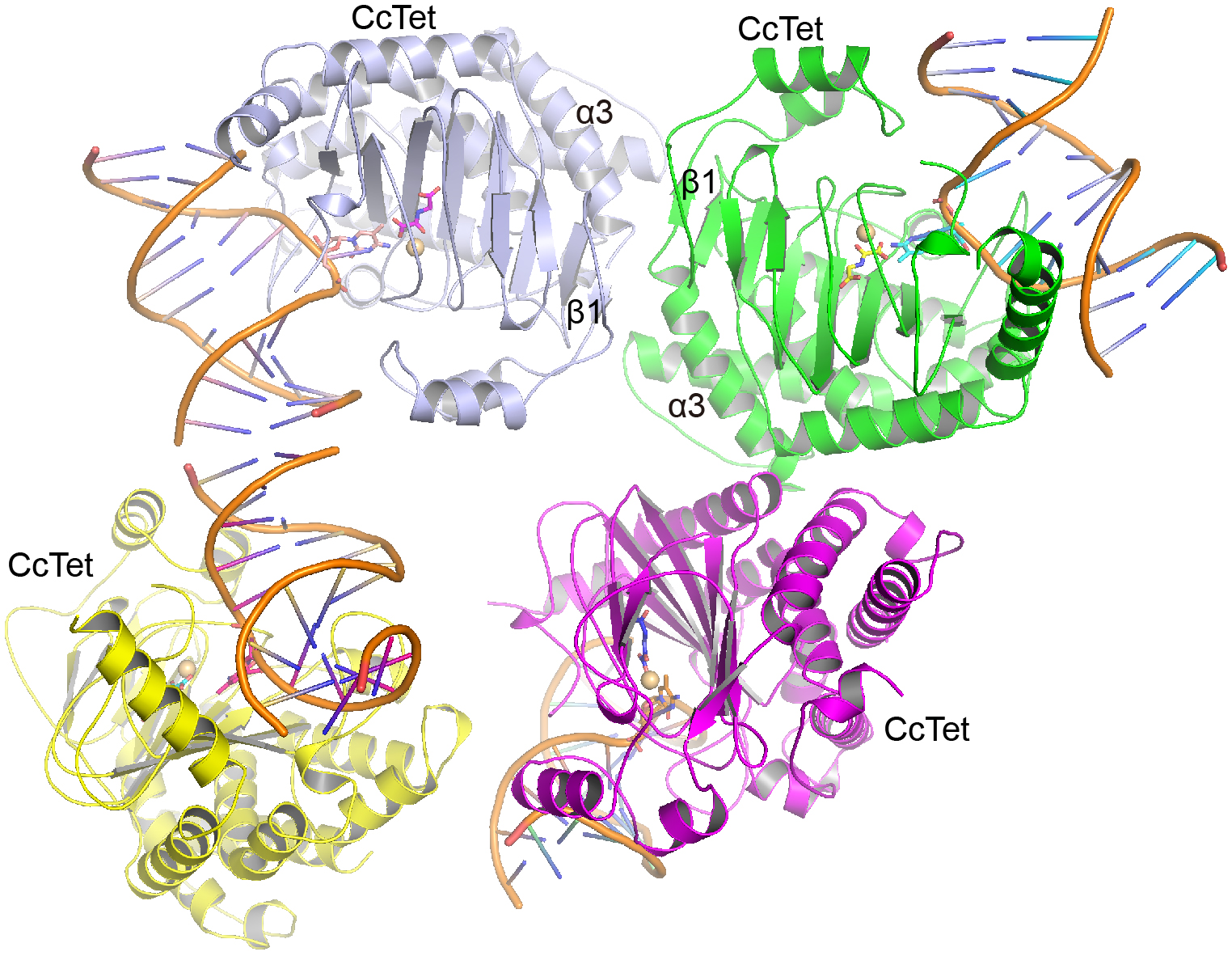
**Supplementary Figure 2.** CcTet–5mC-dsDNA copies observed in the asymmetric unit. Four CcTet–5mC-dsDNA molecules were colored in green, magenta, slate and yellow. The Mn^2+^ ions were shown in golden spheres, and the N-oxalylglycine (NOG) ligands were shown in sticks.

**
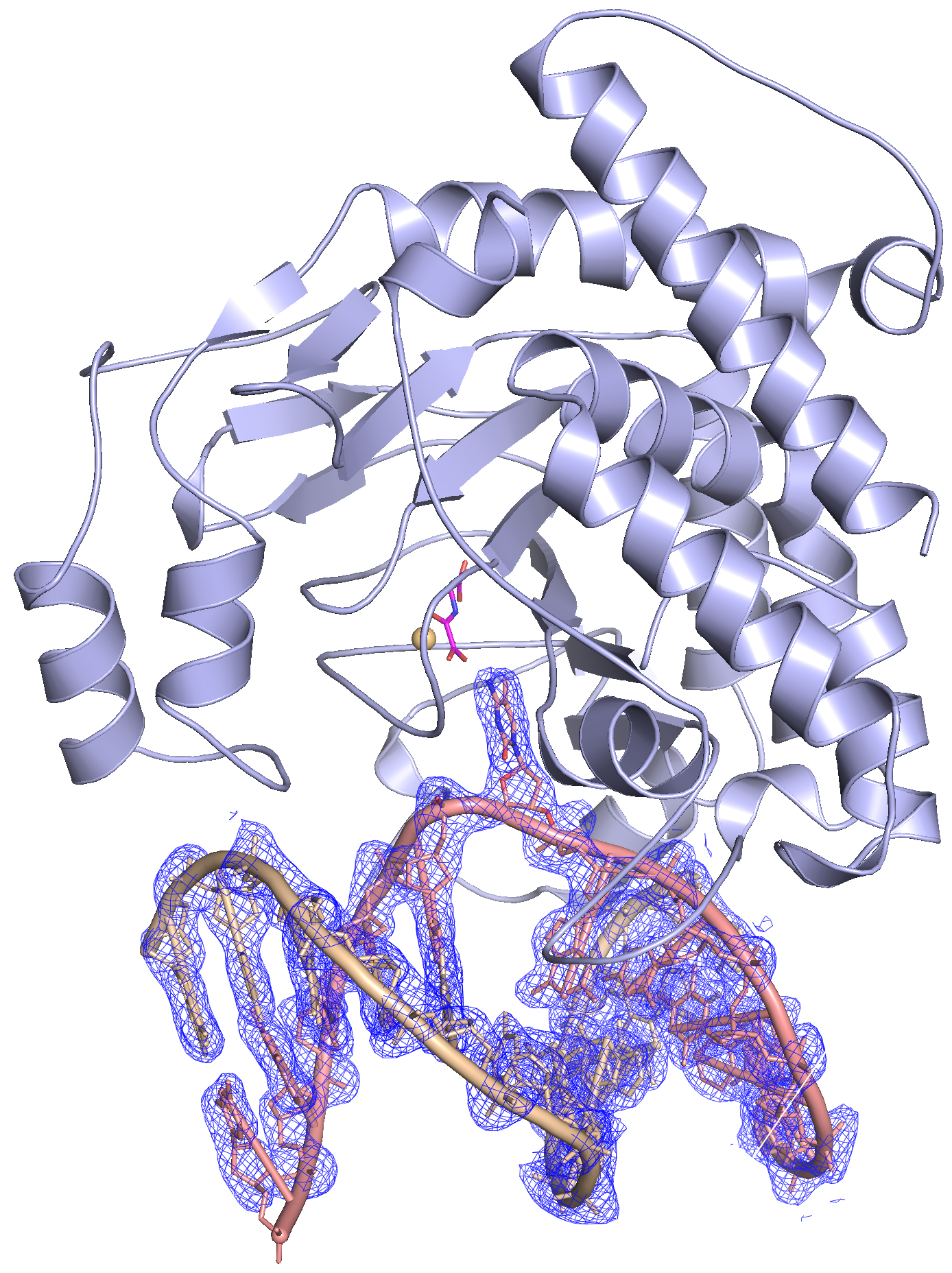
**

**Supplementary Figure 3.** The 2fofc electron density map contoured at 1.0 around the 5mC-dsDNA in the complex structure.

**
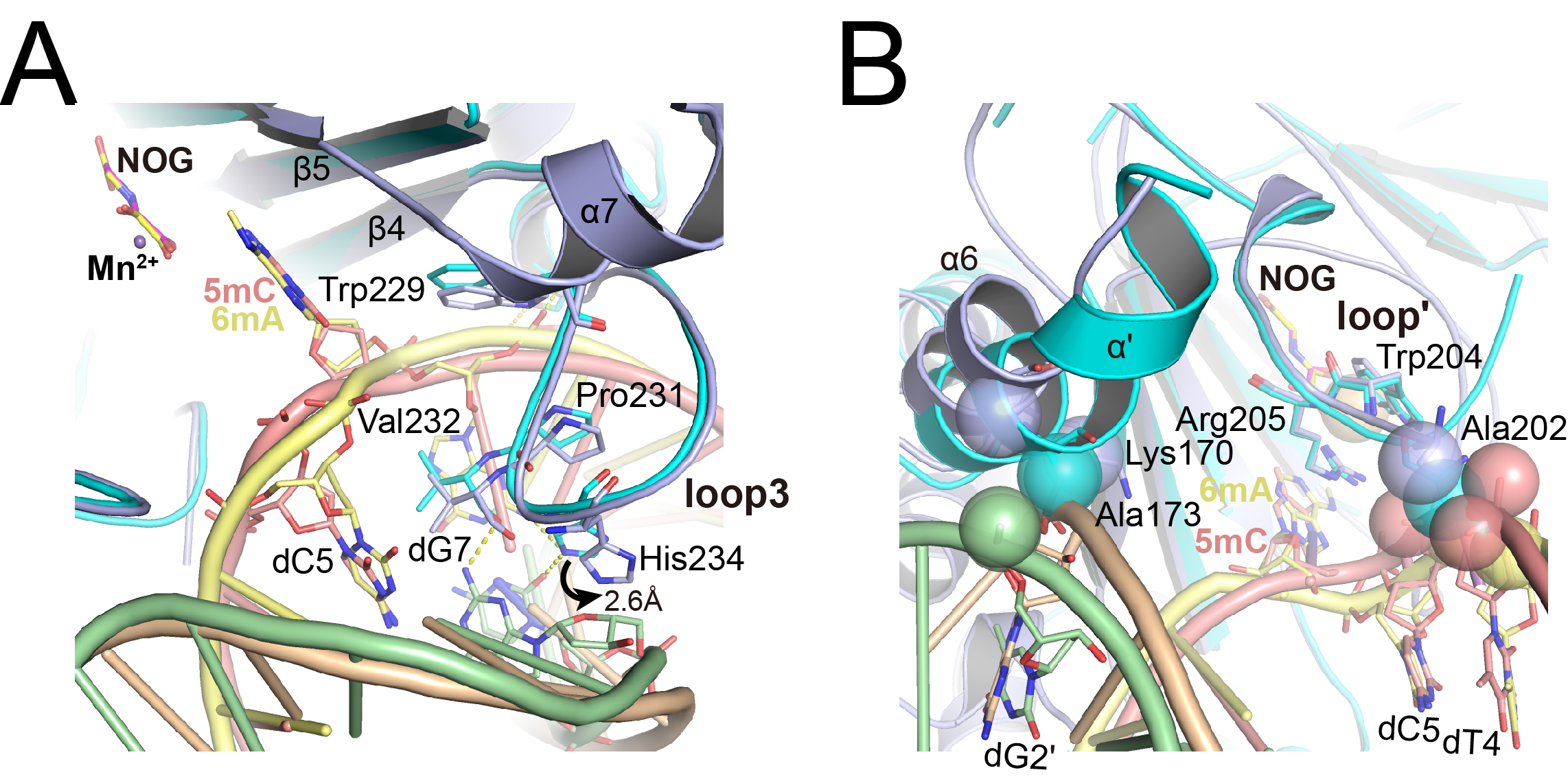
**

**Supplementary Figure 4.** Superposition of the Loop3 (A) and Loop2 (B) regions from CcTet–5mC-dsDNA (slate/wheat/light pink) and CcTet–6mA-dsDNA A (cyan/forest green/yellow) complexes. The black arrow indicates the movement of His234 sidechain upon 5mC-dsDNA binding compared to that of 6mA-dsDNA.

**
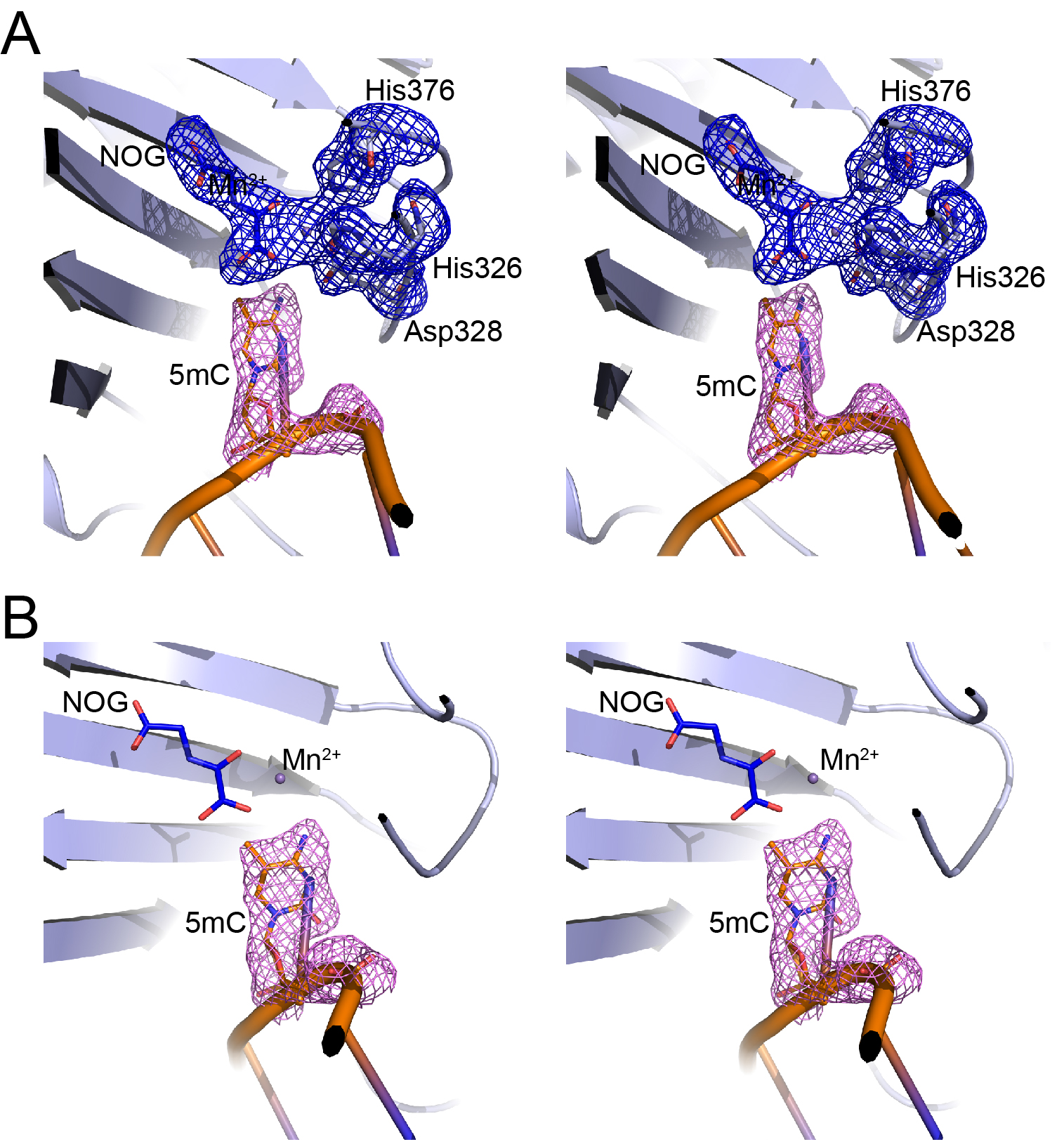
**

**Supplementary Figure 5.** The fofc omit electron density contoured at 3.0σ around 5mC (colored in magenta) and 2fofc electron density contoured at 1.0σ around the three key catalytic residues and NOG and Mn^2+^ ligands (colored in blue).

**
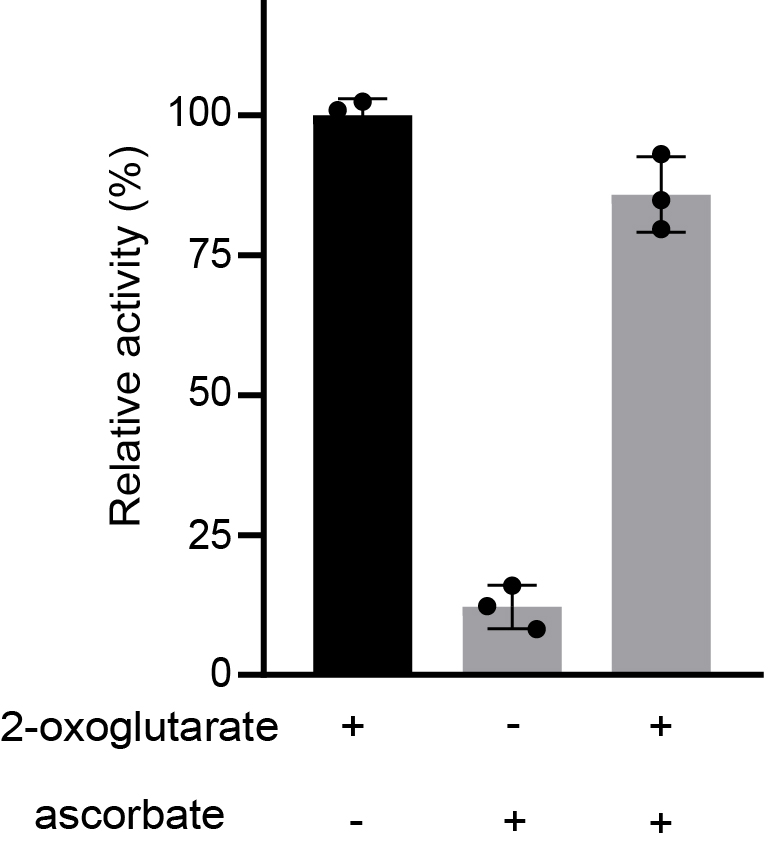
**

**Supplementary Figure 6.** The enzymatic activity of CcTet in the presence and absence of 2-oxoglutarate and L-ascorbate (2mM).
